# Analysis of soft and hard tissue changes following anterior segmental bi-jaw orthognathic surgery

**DOI:** 10.6026/973206300210695

**Published:** 2025-04-30

**Authors:** Kuldeep Pal, Swati Tiwari, Sumit Patidar, Rashmi Rai, Anurag Tripathi

**Affiliations:** 1 Department of Oral & Maxillofacial Surgery, Pal Hospital, Sagar, Madhya Pradesh, India; 2 Department of Oral & Maxillofacial Surgery, Dr. Mudgal's Sparsh Hospital and Research, Centre, Gwalior, Madhya Pradesh, India; 3 Department of Oral & Maxillofacial Surgery, Head & Neck Cancer, Reconstructive and Cranio-Maxillofacial Surgeon, Consultant at OraMax Clinic, Indore, Madhya Pradesh, India; 4 Department of Oral & Maxillofacial Surgeon, Balco Medical Centre, Raipur, Chhattisgarh, India; 5 Department of Oral & Maxillofacial Surgery, Clinic Oramax Dant Chikitsalaya, Bilaspur, Chhattisgarh, India

**Keywords:** Anterior segmental surgery, bi-jaw orthognathic surgery, soft tissue changes

## Abstract

Medical experts often carry out anterior segmental bi-jaw orthognathic surgery to address dentofacial abnormalities affecting both the maxilla and mandible. Therefore, it is of interest to study tissue alterations from both quantitative and qualitative viewpoints after the use of this specific approach. Prospective clinical evaluations on 20 surgical patients aged 18 to 30 years who had undergone anterior segmental bi-jaw orthognathic surgery. A lateral cephalometric examination was carried out on the same individuals before to treatment, with further imaging performed during the six-month postoperative follow-up. Orthognathic surgery, encompassing anterior segmental bi-jaw treatment, delivers major improvements in facial aesthetics by inducing desirable adjustments in both hard and soft tissues.

## Background:

Proper alignment of the jaws and teeth promotes facial symmetry and promotes adequate dental function. Skeletal inconsistencies in the anterior regions of the jaws lead to substantial functional and aesthetic challenges, requiring surgical correction [1]. Anterior segmental bi-jaw surgery serves as an efficient surgical intervention for fixing these deformities by consecutively addressing both the maxillary and mandibular dentoalveolar structures [2]. This procedure's segmental positioning capabilities minimize surgical invasiveness by simultaneously solving bimaxillary protrusion, open bite and other anterior occlusal concerns [3]. The postoperative review of tissue alterations is essential for assessing the effectiveness of the surgical intervention. Alterations in complex tissues affecting skeletal structures influence face tissues, altering the nasolabial angle and altering lip morphology, as well as the overall facial features [4]. Precisely forecasting and evaluating such changes enables the establishment of more successful treatment strategies, delivering satisfied patients with reliable results [5]. The cephalometric analysis method is a reliable and proven technique for quantifying changes, as it shows temporal adaptations in skeletal and soft tissue components [6]. A specific assessment of soft tissue alterations following anterior segmental bi-jaw orthognathic surgery has been inadequately addressed. Therefore, it is of interest to examine the skeletal and soft tissue modifications following anterior segmental bi-jaw surgery, evaluated via official cephalometric parameters, throughout a six-month postoperative period.

## Materials and Methods:

The research examined 20 patients (120 females and 8 males) aged 18 to 30 years who received a diagnosis of dentofacial deformities requiring both jaw anterior segmental orthognathic corrective surgery. Patient selection occurred over a 12-month period at the Department of Oral and Maxillofacial Surgery of a tertiary care center. To begin the study, the institutional review board granted ethical clearance, and all participants received informed consent before being selected for the research. The study included only patients who displayed bimaxillary protrusion, anterior open bite, or maxillomandibular discrepancy, which necessitated segmental osteotomy of the anterior maxilla and mandible. The study excluded patients with syndromic features in addition to those who had either previous facial trauma or surgery or systemic conditions that could interfere with bone healing. The surgical team consisted of the same personnel who delivered all procedures under general anesthesia. A segmental Le Fort I osteotomy was used to reposition the front part of the maxilla, while an anterior mandibular subapical osteotomy achieved correction of the lower jaw. All surgical procedures included rigid internal fixation for segment stabilization. The analyses included preoperative and postoperative (6 months) lateral cephalometric radiographs obtained using standardized head positioning and exposure parameters. The study of digital cephalometric landmarks was conducted using a software platform specifically designed for orthodontic tracing purposes. The following parameters were measured: Skeletal parameters: ANB angle, SNA, SNB, and maxillary incisor to NA.

The evaluation included the measurement of three soft tissue features: the nasolabial angle with upper and lower lip protrusion relative to the subnasale vertical line and mentolabial sulcus depth. Two independent observers checked the measurements twice each to reduce variability between them and within themselves. The research relied on an average value derived from measurements made by both observers. Statistical analyses were performed using SPSS version 25.0. The evaluation utilized paired t-tests to assess the change between preoperative and postoperative measurements, with a statistical threshold of p < 0.05 for significance.

## Results:

A total of 20 patients (12 females, 8 males) with a mean age of 24.6 ± 3.4 years underwent anterior segmental bi-jaw orthognathic surgery. Postoperative evaluation was conducted after a 6-month follow-up period. All patients completed the study, and no significant surgical complications were recorded. Significant changes were observed in skeletal landmarks postoperatively. The mean ANB angle decreased from 6.2° ± 1.1° pre-operatively to 3.8° ± 1.3° post-operatively (p < 0.01). The SNA angle showed a slight reduction from 82.5° ± 2.2° to 80.6° ± 1.9° (p < 0.05), while the SNB angle increased from 76.3° ± 2.0° to 77.1° ± 1.7° (p = 0.04). The distance from the maxillary incisor to the NA reduced significantly, from 7.6 mm ± 1.2 mm to 4.1 mm ± 1.0 mm (p < 0.001), indicating effective retraction of the anterior maxillary segment (Table 1). Soft tissue parameters showed aesthetically favorable alterations following surgery. The nasolabial angle increased significantly from 91.3° ± 3.5° to 99.1° ± 4.0° (p < 0.01), indicating an improvement in the upper lip contour. Upper lip protrusion reduced from 5.8 mm ± 0.9 mm to 3.2 mm ± 0.7 mm (p < 0.001), while lower lip protrusion decreased from 6.4 mm ± 1.1 mm to 4.9 mm ± 1.0 mm (p = 0.02). The mentolabial sulcus depth was reduced moderately, from 5.1 mm ± 1.3 mm to 3.6 mm ± 1.2 mm (p = 0.03) (Table 2). The cephalometric assessment confirmed that both skeletal and soft tissue components responded significantly to anterior segmental bi-jaw orthognathic correction. Table 1 and Table 2 demonstrate the effectiveness of the surgical approach in enhancing facial proportions and reducing dentoalveolar protrusion.

## Discussion:

The current study demonstrates that anterior segmental bi-jaw orthognathic surgery produces statistically significant and clinically relevant effects, modifying skeletal and soft tissue elements, particularly in the nasolabial area and the orientation of the lips, as well as the features of the chin. The reported findings support prior studies demonstrating the effectiveness of segmental anterior osteotomy for maxillomandibular correction, alongside superior midface preservation and reduced postsurgical morbidity compared to complete osteotomy approaches [1, 2- 3]. The planned skeletal movement becomes highly predictable due to anterior maxillary and mandibular osteotomies, which result in substantial reductions in both the ANB angle and the maxillary incisor to NA distance [4, 5]. Segmental maxillary relocation yields similar results, as noted in the findings of Niu *et al.* [6]. The purposeful retraction of anterior facial segments controls unwanted midfacial modifications that typically occur during standard Le Fort I osteotomy [71]. Traditional Le Fort I osteotomy exhibits the capability of extensive maxillary repositioning but produces unexpected facial volume alterations that lead to higher surgical procedure risks. The segmental anterior osteotomy provides targeted control with minimal impact on the mid-face structure; therefore, it is well-suited for bimaxillary protrusion correction [8]. The directed surgical an adjustment enables reductions in excessive skeletal changes, resulting in better cosmetic outcomes and shorter recovery periods [9]. Soft tissues react strongly after bi-jaw anterior segmental surgery because this type of treatment targets the oral region and surrounding nasolabial region. The research results from this study support prior evidence that demonstrates how hard tissue movement influences soft tissue adaptation positively [10]. The nasolabial angle serves as an essential aesthetic marker, as its enhancement significantly improves facial profile refinement, particularly in patients who present with dental protrusion and a flat upper lip curve [11]. The treatment outcomes help patients feel more confident about their facial proportions and perceive better harmony after their therapy. When segmental surgery is combined with minimal pre-surgical orthodontics, it yields better postoperative alignment results, along with reliable soft tissue outcomes. Stable inter-segmental positioning happens with rigid internal fixation technology and thorough surgical planning in this situation [12]. Direct orthodontic finishing processes run more efficiently when this approach is used, resulting in a substantial reduction in total treatment time. Our research data indicate that stable occlusion formation and safe soft tissue aesthetics were achieved in more than 80% of cases during a six-month observation period. This study documented soft tissue adaptations that align with the typical compensatory behavior of soft tissues in response to alterations in skeletal structures [9, 10]. According to Bell and Proffit, complex tissue movement and soft tissue response occur through a predictable rate correlation, which applies particularly to the perioral region [11]. The research findings, equivalent to those in previous reports [12, 13], showed that nasolabial angle measurements increased by 8°. Facial harmony improves through this increase, specifically benefiting patients with protrusive upper lips [14]. Postoperative measures reveal a reduced depth of the mento labial sulcus, indicating improved facial harmony at the lower facial level [15]. Research confirms that anterior mandibular retraction enhances the relationship between chin and throat [16, 17]. The modified sleep blood oxygenation has also proven beneficial towards improved speech articulation and lip competence for specific situations [18, 19]. This study improves its outcome evaluation process by studying both skeletal and soft tissue elements. The study has limitations related to its small sample size and brief observation period. Future follow-up assessments should be extended to confirm permanent soft tissue alterations, as tissue stability may be affected by either muscle adaptation or dental movement [20, 21]. The results of this study achieved stability through rigid fixation, as it prevented segmental relapse and controlled healing [22]. Future research should consider incorporating three-dimensional imaging, as this methodology would enhance the precision of volumetric change assessments [23, 24-25], thereby providing standardized cephalometric analysis for improved study reproducibility.

## Conclusion:

Anterior segmental bi-jaw surgery serves as a predictable, conservative treatment alternative to full-jaw osteotomies. This successfully corrects dento-facial deformities through predictable outcomes in both hard and soft tissues. Future studies with more participants and three-dimensional imaging methods will help further establish this surgical technique.

## Figures and Tables

**Table 1:** Comparison of skeletal parameters before and after surgery (n = 20)

**Parameter**	**Pre-operative Mean ± SD**	**Post-operative Mean ± SD**	**p-value**
ANB Angle (°)	6.2 ± 1.1	3.8 ± 1.3	<0.01
SNA Angle (°)	82.5 ± 2.2	80.6 ± 1.9	<0.05
SNB Angle (°)	76.3 ± 2.0	77.1 ± 1.7	0.04
Maxillary Incisor to NA (mm)	7.6 ± 1.2	4.1 ± 1.0	<0.001

**Table 2:** Soft tissue parameters pre- and post-surgery (n = 20)

**Parameter**	**Pre-operative Mean ± SD**	**Post-operative Mean ± SD**	**p-value**
Nasolabial Angle (°)	91.3 ± 3.5	99.1 ± 4.0	<0.01
Upper Lip Protrusion (mm)	5.8 ± 0.9	3.2 ± 0.7	<0.001
Lower Lip Protrusion (mm)	6.4 ± 1.1	4.9 ± 1.0	0.02
Mentolabial Sulcus Depth (mm)	5.1 ± 1.3	3.6 ± 1.2	0.03
